# Spatiotemporal Clustering of Functional Ultrasound Signals at the Single-Voxel Level

**DOI:** 10.1523/ENEURO.0438-24.2025

**Published:** 2025-02-18

**Authors:** Théo Lambert, Hamid Reza Niknejad, Dries Kil, Gabriel Montaldo, Bart Nuttin, Clément Brunner, Alan Urban

**Affiliations:** ^1^Neuro-Electronics Research Flanders, Leuven 3000, Belgium; ^2^VIB, Leuven 3000, Belgium; ^3^Imec, Leuven 3000, Belgium; ^4^Department of Neurosciences, KU Leuven, Leuven 3000, Belgium; ^5^Department of Neurosurgery, UZ Leuven, Leuven 3000, Belgium

**Keywords:** clustering, correlation, functional ultrasound imaging, single voxel, spatiotemporal analysis

## Abstract

Functional ultrasound (fUS) imaging is a well-established neuroimaging technology that offers high spatiotemporal resolution and a large field of view. Typical strategies for analyzing fUS data comprise either region-based averaging, typically based on reference atlases, or correlation with experimental events. Nevertheless, these methodologies possess several inherent limitations, including a restricted utilization of the spatial dimension and a pronounced bias influenced by preconceived notions about the recorded activity. In this study, we put forth single-voxel clustering as a third method to address these issues. A comparison was conducted between the three strategies on a typical dataset comprising visually evoked activity in the superior colliculus in awake mice. The application of single-voxel clustering yielded the generation of detailed activity maps, which revealed a consistent layout of activity and a clear separation between hemodynamic responses. This method is best considered as a complement to region-based averaging and correlation. It has direct applicability to challenging contexts, such as paradigm-free analysis on behaving subjects and brain decoding.

## Significance Statement

The application of spatiotemporal clustering at single-voxel resolution for functional ultrasound (fUS) signal analysis significantly enhances sensitivity in comparison to conventional methods, such as region-based averaging or event correlation. Conventional approaches frequently rely on predefined atlases or specific experimental conditions, which inherently restrict spatiotemporal resolution. In contrast, single-voxel clustering optimizes the potential of fUS, facilitating the detection of intricate activity patterns throughout the brain without the necessity for prior assumptions. This approach enables more precise differentiation of hemodynamic responses and more reliable activity mapping. It is particularly advantageous in complex or paradigm-free studies, offering a high-resolution alternative to standard techniques.

## Introduction

Since its initial development in 2011, the functional ultrasound (fUS) imaging technology (Macé et al., 2011, 2013) has evolved from anesthetized to freely moving animals (Sieu et al., 2015; Urban et al., 2015), from single plane (Macé et al., 2011; Urban et al., 2014) to brain scans (Gesnik et al., 2017; Macé et al., 2018; Brunner et al., 2023) and volumetric recordings (Rabut et al., 2019; Brunner et al., 2020), and from rodents to humans (Demene et al., 2016; Soloukey et al., 2019). Despite this, analytical strategies have progressed at a slower pace, and developments are still needed to fully exploit the potential of the technology. In particular, the analysis of fUS data often relies on a standardized pipeline of registration and segmentation, followed by atlas-based region averaging (Macé et al., 2018; Brunner et al., 2020; Nouhoum et al., 2021) or voxel-to-voxel correlation analysis (Urban et al., 2015; Rungta et al., 2017; Rau et al., 2018; Soloukey et al., 2019).

Although these analyses quickly produce an interpretable visualization, they have several limitations. First, atlas-based region averaging masks spatial variations in hemodynamic activity. It therefore results in the mixing of inactive and active voxels, leading to an underestimation of the true activity or signals arising from different neural processing. Second, the outcome of correlation-based analyses is influenced by the choice of temporal window, which is often biased by expectations of evoked responses. This approach fails to account for brain activity changes occurring during the pre-stimulus period (e.g., task preparation, stimulus anticipation, which frequently occurs when stimuli are repeated multiple times, or pre-motor activity) as well as poststimulus activity (e.g., variations in behavioral responses resulting from delayed neuronal responses associated with cognitive control and decision-making processes). Finally, it does not reflect the temporal dynamics of the signals, as it assesses how well the data fit a square pulse, regardless of the differences between time series.

Thus, the spatial and temporal resolution of fUS is not fully exploited by these techniques, potentially missing important information. This point is further supported by Macé et al. (2018) who mapped the hemodynamic responses (HR) after visual stimulation in >180 brain regions. The presence of differently shaped responses to the same stimulus suggests that neural computations are reflected in the HR and calls for further investigation at a finer scale. This is particularly relevant when considering the correlation of the signal with spiking activity in groups of voxels (Aydin et al., 2020; Nunez-Elizalde et al., 2022).

Therefore, there is a need for a new approach that complements region averaging and correlation analysis by exploiting the high spatiotemporal resolution of fUS in an unbiased manner. In this paper, we propose a spatiotemporal clustering of single voxels, the smallest unit from the µDoppler image, to address this need. Spatiotemporal clustering is a subfield of data mining that has become popular in neuroscience, especially in large-scale imaging, due to its exploratory power and broad applicability (Smolders et al., 2007; Gonzalez-Castillo et al., 2012; Zhang et al., 2014). Its advantages include the limited number of assumptions about HR properties while avoiding the bias introduced by the stimulus pattern. It is important to note that signal intensity in single voxels or paired voxels has been used in several studies as a quality control measure for fUS (e.g., assessing SNR and temporal stability; Rungta et al., 2017; Bimbard et al., 2018; Aydin et al., 2020; Landemard et al., 2021); however, it has not yet been applied to large-scale analyses.

To investigate these methods, we used a dataset consisting of visually evoked hemodynamic activity in the superior colliculus (SC) of awake mice. The mouse SC is a midbrain structure that integrates sensory information, particularly visual information, and plays a major role in a variety of behaviors, including defensive responses to threatening stimuli (Cang et al., 2018; Ito and Feldheim, 2018) and decision-making (Basso and May, 2017). It receives direct input from >85% of retinal ganglion cells, and its layered organization, morphological cell types, circuits, and connections have been extensively studied (Basso and May, 2017; Benavidez et al., 2021). By modulating the luminance contrast of the stimulus between 1 and 100%, we induced different spatiotemporal distributions of HR within the layers of the SC.

On this dataset, we compared region averaging, correlation, and single-voxel clustering for their ability to accurately report the contrast modulation effect on HR. In particular, the ability of each method to detect subtle HR at low luminance contrast was evaluated. We demonstrated that single-voxel clustering generates full-resolution activity maps, enabling the efficient segmentation of active brain regions and offering a detailed, fine-grained representation of the activity distribution. We also discussed the advantages and limitations of each approach, providing the community with a perspective on the trade-off between dimensionality and comprehensiveness. Complementing the signal analysis toolbox of fUS users by providing an open-source software, single-voxel clustering can be applied in many contexts, including paradigm-free analysis, atlas-free investigation, and brain decoding.

## Materials and Methods

### Animals

The presented research was performed in accordance with the Belgian and European laws, guidelines, and policies for animal experimentation, housing, and care (004-2014/EEC, 240-2013/EEC, 252 2015/EEC). All experimental procedures were approved by the Ethical Committee for Animal Experimentation of the KU Leuven. Five male adult (2–4 months old) C57BL6 mice were used for the experiments. Prior to surgery, the mice were kept in group cages in a 12 h dark/light cycle environment at a constant temperature of 21°C. After the surgery, the mice were individually housed in an enriched cage with *ad libitum* access to food and water. All experiments were conducted during the light phase of the animal's activity cycles.

### Surgical procedure

The procedure detailed in Brunner et al. (2021) was strictly followed. General anesthesia was induced by a mix of ketamine (100 mg/kg; Nimatek, Dechra) and medetomidine (1 mg/kg; Domitor, Orion Pharma) injected intraperitoneally. The depth of anesthesia was regularly confirmed by the absence of a pedal reflex when pinching the paw of the animal. Once deep anesthesia was achieved, the mouse head was fixed in a stereotactic frame and the body set in prone position on a homeothermic blanket used to maintain the body temperature at 36.5 ± 0.5°C. Eye ointment was applied to prevent dehydration of the eye (Duratears, Alcon). Subsequently, the top of the animal's head was shaved, cleaned, and wiped with an iso-betadine solution using a sterile foam. Next, the skin was incised and removed to expose the dorsal part of the skull. The lateral and posterior muscles were retracted. Anterior-posterior coordinates were measured from the bregma reference point. A stainless-steel headpost was fixed to the skull using dental cement (Super-Bond C&B, Prestige-dental). Then, a cranial window covering both left and right hemispheres from the bregma −2 to −6 mm and ±5 mm from the midline was made with a micro drill. Finally, the cranial window was covered and protected with silicone rubber (Body Double-Fast Set, Smooth-On). The anesthesia was reversed using atipamezole (1.0–2.5 mg/kg, i.p.; Revertor, Vibrac). The mouse was medicated with painkiller (buprenorphine 0.2 mg/kg, i.p.), antibiotic (cefazolin 15 mg/kg, i.p.), and anti-inflammatory (dexamethasone 0.1 mg/kg) drugs and allowed to recover on in its home cage placed on a heating pad. Before starting the habituation to the handler, the experimental room, and the head fixation system, the mouse received postoperative care (painkiller, antibiotic, and anti-inflammatory drugs) and recover for 7 d.

### Head fixation and habituation

After recovery, mice were trained to be head-fixed while awake by fixing the headpost to the animal holder and then installed in the experimental setup. The period of fixation was progressively increased from 5 min to 2 h. A 5% sucrose solution (Sigma-Aldrich) was used to reward the mice during the training sessions.

### Functional ultrasound imaging sequence

The imaging procedure used was adapted from the sequence for fast, whole-brain functional ultrasound imaging (Macé et al., 2018; Brunner et al., 2021). The imaging scanner is composed of a linear ultrasonic transducer (15 MHz, 128 elements, L22-14v, Vermon) connected to 128-channel emission-reception electronics (Vantage, Verasonics) that are both controlled by a high-performance computing workstation (fUSI-2, AUTC). The transducer was motorized (T-LSM200A, Zaber Technologies) to allow anteroposterior scanning of the brain. Imaging is performed on an antivibration table to minimize external sources of vibration. Each coronal µDoppler image is 12.8 mm width and 9 mm depth and comprises 300 compound images acquired at 500 Hz. Each compound image is computed by adding five plane-wave angles (−6, −3, 0, 3, 6°; three averages per angle, 500 Hz rate). The blood signal was extracted from 300 compound images using a single value decomposition filter and removing the 30 first singular vectors. The µDoppler image is computed as the mean intensity of the blood signal in these 300 frames that is an estimator of the cerebral blood volume (Macé et al., 2011, 2013; Montaldo et al., 2022). This sequence enables a temporal resolution of 0.6 s, an in-plane resolution of 100 × 110 µm, and an off-plane (thickness of the image) of 300 µm. The intensity value of a voxel at a given time was calculated as *I*(*x*,*y*) = *A*(*x*,*y*,*t*)^2^, where *I* is the power Doppler intensity, *x* and *y* are the coordinates of a given voxel in a given plane, *A* is the amplitude of the compound images after filtering, and *t* is the time.

### fUS imaging recording setup

The awake mouse was head-fixed on the platform with the body set in a cylindrical holder. The silicone cap was removed, and the cranial window was covered with a thin layer of 2% agarose to reduce brain movement. Acoustic gel (Unigel, Asept) was applied on the agarose to allow an efficient acoustic coupling. The ultrasound probe was lowered down to a position roughly 3 mm from the brain. The exact location of the superior colliculus was identified by performing a functional scan. The acquisition of the functional data was controlled and synchronized using custom script written in MATLAB.

### Visual stimulus

Visual stimuli were presented on a 32 inch LCD monitor (Samsung S32E590C, 1,920 × 1,080 pixels resolution, 60 Hz refresh rate, maximum luminance of 350 cd/m^2^) positioned at an angle of 45° to the mouse head, 20 cm from the left eye, so that the screen was covering 100° of azimuth and 70° of elevation of the left visual field. Visual stimuli were presented on a gray background (50% luminance) using PsychoPy (Peirce et al., 2019). The stimulus consisted of a full screen checkerboard with a spatial frequency of 0.05 cycles/degree. Each trial started with the presentation of a gray background for a 10 s baseline period, before the stimulus was triggered. The checkerboard flickered at 5 Hz for 4 s and is followed by a 20 s poststimulation period with a gray background. The contrast conditions were set at 1, 2, 3, 5, 10, 30, 50, 90, and 100% luminance contrast and presented in this order. They were defined using the Michelson contrast formula. Each fUS recording session consisted of 450 trials (50 trials/condition).

### Registration and segmentation

µDoppler images were manually aligned to the Allen mouse brain Common Coordinate Framework (CCF; Wang et al., 2020) using an open-access software (Brunner et al., 2021). The µDoppler image was positioned and aligned in the CCF using anatomical landmarks (vessels and sinus) and morphological features (cortical curvature, hemispheres) following the recommended procedure (Brunner et al., 2021). The cross section was then interpolated to the atlas resolution (50 µm^3^) by nearest neighbor interpolation. Duplicate values were removed during model fitting to prevent a statistical imbalance. Translations and scaling in the *x*- and *z*-axes and in-plane rotations were then estimated and stored in a transformation matrix. This matrix was finally used to rigidly transform the data at each time point, yielding CCF labels for each fUS voxel. This procedure has been conducted using PyfUS software features.

### Quantification and statistical analysis

Using PyfUS software, we computed the following parameters for the hemodynamic responses presented in this manuscript (Lindquist et al., 2009; Hirano et al., 2011; Urban et al., 2014):

1. Peak amplitude (Δ*I* in %): the maximum amplitude value reached during the stimulus presentation.
2. Area under the curve (AUC in arbitrary unit): the integral of the amplitude variation during the stimulus presentation, computed using Simpson's estimator (scipy package, v1.7.3, “simpson” function).
3. Time to peak (TTP in seconds): The time point corresponding to the first significant change in the derivative of the signal following the onset of the stimulus.
4. Response length (RL in s): the last time point of the longest sequence of negative derivatives after stimulus onset.
5. Full-width at half-maximum (FWHM in s): length of the longest sequence of amplitude values above half the peak amplitude.
6. Time point of half-maximum (TPHM in %): first time point associated with a signal value higher than half the peak amplitude after stimulus onset.

A low pass filter (order 2; cutoff frequency, 1 Hz) was applied before computing the time to peak and the response length.

### Single-voxel clustering

The PyfUS software was used to apply a principal component analysis (PCA; Gewers et al., 2022) to the dataset, regrouping the single-voxel signals of the superior colliculus from all animals and contrast conditions. The 12 first components of the PCA were kept as features, corresponding to 50% of explained variance (Extended Data Fig. 1-1). This lower-dimension dataset was then clustered using the Elkan version of the *K*-Means algorithm (Hamerly, 2010). The number of clusters was determined using the elbow method (Yuan and Yang, 2019) on the inertia (Extended Data Fig. 1-1). Once set, the algorithm was run with 100 different initializations and the iteration with the lowest inertia was kept as final model. The robustness of the clustering was evaluated by performing the clustering procedure over 10 iterations and calculating the average similarity between pairs of cluster maps across these iterations. Similarity was defined as the proportion of voxels assigned the same cluster label for a given pair of maps, with clusters reordered based on amplitude. The implementations of both PCA and K-Means were performed using the “scikit-learn” library (v1.0.1; Pedregosa et al., 2012) in Python (v3.7.7).

### Data availability

Several steps of the analysis procedure were performed using the PyfUS software. The PyfUS software used and described in the paper is publicly available online at https://github.com/OpenfUS/PyfUS. The code is available as Extended Data. The dataset is available at https://doi.org/10.5281/zenodo.14534340.

10.1523/ENEURO.0438-24.2025.d1 Extended Data Download Extended Data, ZIP file.

## Results

**Figure 1.**
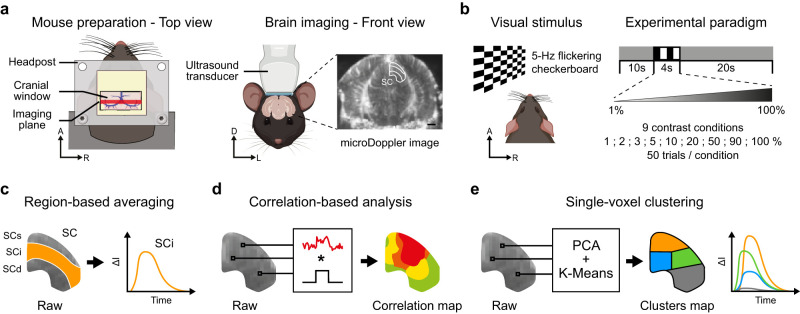
Experimental setup and analysis methods. ***a***, Schematic representation of the acquisition setup. Left, The mouse was prepared with a cranial window providing access to the imaging plane. Right, The 15 MHz ultrasound transducer was positioned and aligned over the superior colliculus (SC; bregma −4.0 mm), outlined in white on a µDoppler image. Scale bar, 1 mm. A, anterior; D, dorsal; L, left; R, right. ***b***, During the acquisition, the mouse was subjected to a 5 Hz flickering checkerboard stimulus covering the full visual field of the left eye. The 4 s stimulus was presented 10 s after baseline and followed by a 20 s poststimulus period, each corresponding to a gray background screen view. Nine contrast conditions were used for the stimulus, and 50 trials per condition were acquired. A, anterior; R, right. ***c***, The region averaging procedure consists of averaging all the voxels belonging to a given region [e.g., superficial (SCs), intermediate (SCi), and deep layer of the superior colliculus (SCd); SCi is highlighted in orange] to obtain a single temporal trace representing the mean activity of the region. Δ*I* denotes the signal amplitude. ***d***, The correlation analysis consists of computing the Pearson’s correlation coefficient between the temporal trace (in red) of each individual voxel and a square window (in black) replicating the stimulus pattern. It results in a correlation map, in which each voxel is color coded according to its correlation coefficient. ***e***, The single-voxel clustering procedure first involves reducing the dimensionality of the single-voxel temporal traces using a principal component analysis (PCA; Extended Data Fig. 1-1). The output is then fed into a K-Means clustering algorithm (Extended Data Fig. 1-1). The process results in (1) a cluster map in which each voxel is color coded according to its cluster attribution and (2) the temporal traces associated with each cluster.

In this study, we used a dataset comprising *n* = 5 mice. Each one was subjected to a cranial window providing access to the superior colliculus (SC) and imaged with a 15 MHz linear ultrasound transducer (Fig. 1*a*). Mice were presented with a 5 Hz flickering checkerboard stimulus of 4 s. The luminance contrast was modulated between 1, 2, 3, 5, 10, 20, 50, 90, and 100% (Fig. 1*b*). Each stimulus was preceded by a 10 s period and followed by a 20 s period, during which a gray screen was presented. The data were registered to the Allen CCF (Wang et al., 2020) for extracting the voxels belonging to the different layers of the SC (SCs, superficial; SCi, intermediate; SCd, deep). This step is required for comparing on the same basis region-based averaging with correlation-based analysis and single-voxel clustering, as detailed below.

10.1523/ENEURO.0438-24.2025.f1-1 Figure 1-1 **Parameters selection for single-voxel clustering**. **a.** Cumulated explained variance ratio of the PCA with respect to the number of principal components. The value 12 was selected, retaining 50% of the explained variance (dashed line). **b.** Evolution of the inertia with respect to the selected number of clusters. The inflexion point of the curve was used to select K = 5 clusters. Download Figure 1-1, TIF file.

The region averaging procedure consists of selecting all the temporal traces of the voxels belonging to a given region or subregion and averaging them to obtain a single temporal trace representing the region activity (Fig. 1*c*). To perform the correlation-based analysis, the Pearson’s correlation coefficient between (1) a temporal window matching the stimulus onset/offset and (2) the temporal trace of single voxels is computed, resulting in the *R*^2^ score and presented as a correlation map (Fig. 1*d*). Such a map can further be binarized applying a Fisher transform, resulting in the set of voxels whose correlation with the stimulus is statistically significant at a given confidence level. Importantly, in the context of a single stimulus pattern, the Pearson’s correlation coefficient is equivalent to a general linear model with normalized data. Finally, the clustering of single voxels is achieved by reducing the dimensionality of the temporal traces of single voxels with a PCA before applying a *K*-Means algorithm. Recording sessions from all mice and contrast conditions were pooled together as the input dataset. It results in a map in which each voxel is color coded according to its cluster and the associated temporal traces (Fig. 1*e*).

### Region-based averaging

**Figure 2.**
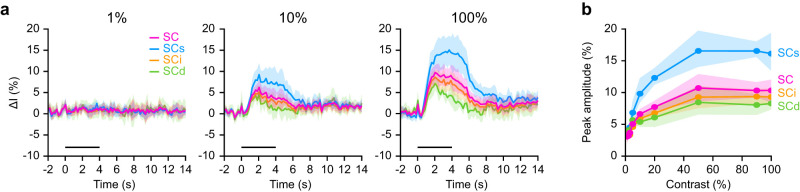
Region-based averaging analysis. ***a***, Average hemodynamic response curves (Δ*I* in %) in response to 1, 10, and 100% luminance contrast stimulation (left to right) for the different layers and full SC (50 trials/mouse, *n* = 5). The stimulus period (0–4 s) is depicted by the horizontal black line. Error bands denote the standard deviation. ***b***, Average peak amplitude (in %) in function of the luminance contrast for the different layers and the full SC. The peak amplitude is defined as the maximum signal intensity reached during the stimulus presentation. Error bands represent the 95% confidence interval. SC, superior colliculus (magenta); SCs, superficial layer of the SC (cyan); SCi, intermediate layer of the SC (orange); SCd, deep layer of the SC (green).

We first investigated the outcome of the region-based averaging procedure across the different layers of the SC. The increase in the luminance contrast induced an increase in HR amplitude in all layers, with higher values in SCs, followed by SCi and SCd (for 1, 10, and 100%; Fig. 2*a*). The time courses of the HR were marked by a steep increase of the signal, up to a plateau, and was followed by a slow decrease back to baseline level, with seemingly longer return-to-baseline period at higher contrasts. The shape in the SCi was similar to the average on the full SC, while the SCs HR display longer plateau stage. On the contrary, SCd HR did not exhibit a plateau during or after stimulation.

The contrast–response curves were computed layer-wise using the peak amplitude value reached during the stimulus presentation (Fig. 2*b*). In general, the contrast modulation had a clear effect in all layers, with an increase in peak amplitude together with the increase in luminance contrast. The contrast–response curves resemble a logarithmic form and reach a plateau from 50% luminance contrast onward. The highest peak amplitude was found in the SCs (16.5 ± 1.6% at 90% contrast; mean ± SD, *n* = 5), followed by the SCi (9.4 ± 1.6% at 90% contrast; mean ± SD, *n* = 5) and SCd (8.2 ± 1.2% at 100% contrast; mean ± SD, *n* = 5).

The conclusions of the region-based averaging analysis are (1) the peak amplitudes are ranked in the order SCs, SCi, and SCd, (2) the peak amplitude increases with contrast up to 50%, and (3) HR tend to be longer toward superficial layers.

### Correlation-based analysis

**Figure 3.**
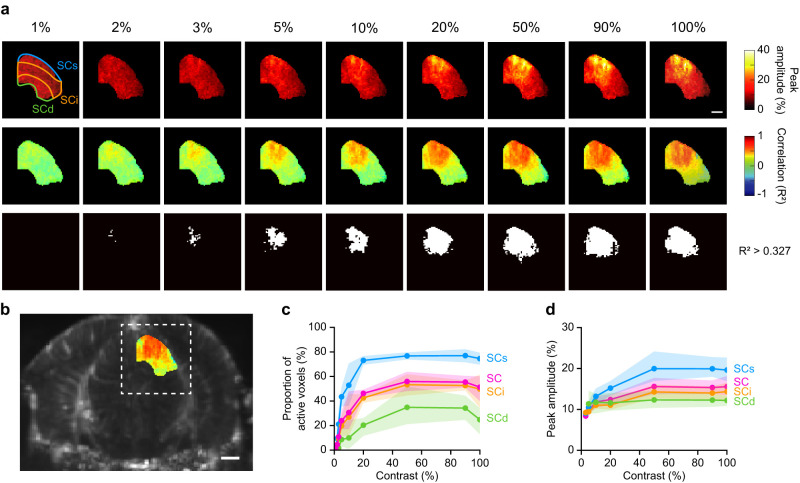
Correlation-based analysis. ***a***, Average peak amplitude (%, top row) and correlation (*R*^2^, middle row) maps for all contrast intensities, at the single-voxel level. Correlation maps were binarized with a threshold of 0.327 (bottom row), corresponding to the statistical significance level *p* < 0.01. Scale bar, 0.5 mm. ***b***, µDoppler image overlaid with the correlation map at 100% contrast for mouse 5 (for all mice, see Extended Data Fig. 3-1). Scale bar, 1 mm. ***c***, Layer-wise change in the proportion of active voxels with luminance contrast. The proportion of active voxels is defined as the ratio between the number of voxels at *R*^2^ > 0.327 and the total amount of voxels in the structure. Error bands represent the 95% confidence interval. ***d***, Layer-wise peak amplitude extracted in the active voxels of each structure in function of the luminance contrast. The plot illustrates the peak amplitude extracted from active voxels in each structure of the superior colliculus as a function of luminance contrast. Voxels originating from active areas representing <1% of the structure were excluded. Error bands represent the 95% confidence interval. SC, superior colliculus (magenta); SCs, superficial layer of the SC (cyan); SCi, intermediate layer of the SC (orange); SCd, deep layer of the SC (green).

The correlation-based analysis was then performed on the same dataset. The intermouse averages of the HR peak amplitude and the resulting correlation maps are displayed for the different contrasts on Figure 3*a* (top and middle row, respectively). For visualization purposes, the correlation map of one mouse at 100% luminance contrast was overlaid on the associated µDoppler image on Figure 3*b*. Depending on the individual, correlation become apparent starting from 2 to 3% of luminance contrast (Extended Data Fig. 3-1). The maps exhibited a similar increase in spatial spread for the signal amplitude and the correlation value (*R*^2^) over the medial and centromedial parts of the SC, in function of the stimulus contrast.

10.1523/ENEURO.0438-24.2025.f3-1 Figure 3-1 Spatial maps across animals and contrasts Spatial maps of the superior colliculus (SC) across all contrast intensities and all the mice (n = 5) used in this work, resulting from peak amplitude computation (left), correlation (center left), correlation with threshold (center right) and single-voxel clustering (right). Scale bar: 0.5  mm. Download Figure 3-1, TIF file.

The correlation maps were binarized using the threshold *R*^2^ > 0.327 (Fisher transform, *p* < 0.01; Fig. 3*a*, bottom row), and the active area was defined as the set of voxels with a correlation coefficient higher than this threshold. The proportion of active voxels increases with the luminance contrast, following a logarithmic shape that is not layer specific (Fig. 3*c*). The proportion of active voxels in SCi and SCd exhibit a plateau from 50% contrast while the plateau starts at 20% luminance contrast for the SCs. This proportion ranges between 0 and 2% at 1% luminance contrast for all layers of the SC and goes up to 77.0 ± 6.2%, 53.3 ± 9.7%, and 34.8 ± 17.2% for SC, SCs, SCi, and SCd, respectively (Fig. 3*c*).

We further computed the amplitude of the averaged signal within the active area for the full SC and layers. The variability in peak amplitude at low-contrast conditions is high as it corresponds to the smallest proportion of active voxels in each region. The resulting amplitude-to-contrast curves increase up to a plateau starting at 50% contrast for the SC, SCs, and SCi and at 20% contrast for SCd with maximum peak amplitude of 15.6 ± 2.3%, 19.9 ± 2.8%, 14.3 ± 2.6%, and 12.3 ± 2.5%, respectively, for SC, SCs, SCi, and SCd (Fig. 3*d*).

The conclusions of the correlation-based analysis are similar to those from the region-based averaging, with additional insights: (1) the proportion of active voxels is ranked from SCs (high), SCi (medium), to SCd (low); (2) the proportion of active voxels in the layers stops increasing at lower contrast than the peak amplitude in the SCs (i.e., 20 vs 50% luminance contrast), indicating that the increase in peak amplitude from 10 to 20% contrast arises from higher amplitude levels in already active voxels; and (3) even at high luminance contrast, the entire layers are not becoming active.

### Single-voxel clustering

**Figure 4.**
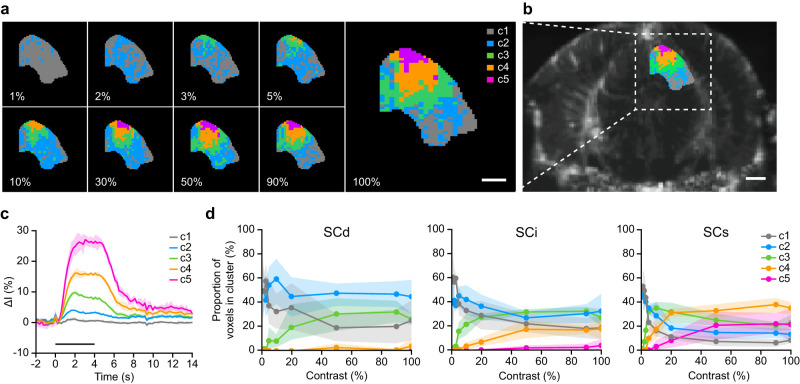
Single-voxel clustering analysis. ***a***, Representative change of clustering-based activity maps in the SC associated with the change in luminance contrast (from 1 to 100%). Each voxel is color coded based on its cluster attribution. c1, gray; c2, cyan; c3, green; c4, orange; c5, purple. Scale bar, 0.5 mm. ***b***, µDoppler image overlaid with the cluster map at 100% contrast for mouse 5 (for all mice, see Extended Data Fig. 3-1). Scale bar, 1 mm. ***c***, Average temporal traces for each cluster (c1–c5). Error bands denote the standard deviation. The stimulus period (0–4 s) is depicted by the horizontal black line. ***d***, Layer-wise change in the proportion of voxels attributed to each cluster (c1–c5), in function of the luminance contrast (1–100%). Error bands denote the 95% confidence interval. SC, superior colliculus (magenta); SCs, superficial layer of the SC (cyan); SCi, intermediate layer of the SC (orange), SCd, deep layer of the SC (green).

The clustering process (see Materials and Methods) resulted in five distinct clusters within the SC. Each voxel was color coded based on its cluster assignment (Fig. 4*a*,*b*; Extended Data Fig. 3-1). The different contrast intensities exhibited different compositions of clusters that appeared in the order of c1/c2, c3, c4, and finally c5. The robustness of the clustering outcome was validated by evaluating the similarity of cluster maps across iterations, yielding an average similarity of 95.9 ± 4.8% (see Materials and Methods). Interestingly, the voxels belonging to the same cluster are spatially connected, especially from 3% luminance contrast onward. The voxels switching the most from one cluster to another (e.g., c3–c4) are located in the medial and centromedial parts of the SC.

The peak amplitude and the area under the curve (AUC) appeared to be the main differentiating features between the clusters [one-way ANOVA for mean peak amplitude (1.4–27.6%, standard deviation 0.3%, *p* < 0.0001) and mean AUC (0.1–41.4, standard deviation 0.4, *p* < 0.0001)]. Table 1 provides an overview of the HR metrics conventionally used in the literature (Lindquist et al., 2009; Hirano et al., 2011; Urban et al., 2014).

**Table 1.** Per-cluster hemodynamic responses characteristics

Cluster	TTP (s)	RL (s)	Peak amplitude (%)	AUC (a.u.)	FWHM (s)	HMTP (s)
c1	3.0 ± 3.0	5.9 ± 4.2	1.3 ± 0.0	0.1 ± 0.3	0.6 ± 0.3	0.3 ± 0.7
c2	1.8 ± 0.0	7.3 ± 4.1	3.9 ± 0.1	6.9 ± 0.2	4.2 ± 0.4	0.4 ± 0.6
c3	1.9 ± 0.1	7.1 ± 0.6	9.7 ± 0.5	13.5 ± 0.5	4.4 ± 0.2	1.2 ± 0.1
c4	2.9 ± 1.1	7.4 ± 0.5	16.9 ± 0.1	25.5 ± 0.9	4.8 ± 0.2	1.1 ± 0.1
c5	3.2 ± 0.8	8.1 ± 1.3	27.6 ± 1.1	41.4 ± 1.6	5.0 ± 0.2	1.1 ± 0.1

TTP, time to peak; RL, response length; AUC, area under the curve; FWHM, full-width at half-maximum; HMTP, half-maximum time point. Values are mean ± SD for *n* = 5 mice and computed independently of the luminance contrast.

Apart from cluster c1 (Fig. 4*a*, gray), all clusters exhibit a substantial increase in the signal amplitude following the stimulus presentation (Fig. 4*c*). Average peak amplitude of 3.9 ± 0.1%, 9.7 ± 0.5%, 16.9 ± 0.7%, and 27.6 ± 1.1% (mean ± SD, *n* = 5) was measured for clusters c2–c5, respectively. The cluster distribution significantly varied across individuals in terms of (1) presence/absence of clusters at a given contrast (e.g., cluster c3 at 1–2% contrast for mice 1 and 5), (2) number of voxels assigned to a cluster, and (3) location (Extended Data Fig. 3-1).

The layer-wise distribution of each cluster, independently of the contrast, is presented in Table 2. Clusters c4 and c5, corresponding to the highest peak amplitude, are almost exclusively present in the SCs and SCi. The SCs accounts for 59.3 ± 11.1% of the voxels from c4, the second most active cluster, and 88.7 ± 13.4% from c5, the most active cluster. The other three clusters (c1–c3) are mostly observed in the SCi first, then in the SCs, and finally in the SCd.

**Table 2.** Layer-wise distribution of clusters

Cluster	Proportion of voxels in layers of the superior colliculus (%)		
	Superficial layer—SCs	Intermediate layer—SCi	Deep layer—SCd
c1	19.9 ± 1.9	57.5 ± 2.0	22.6 ± 2.0
c2	21.0 ± 1.6	51.3 ± 3.0	27.6 ± 2.7
c3	32.6 ± 3.7	51.3 ± 3.1	16.1 ± 4.1
c4	59.3 ± 11.1	39.0 ± 9.5	1.6 ± 2.3
c5	88.7 ± 13.4	11.3 ± 13.4	0.0 ± 0.0

SC, superior colliculus; SCs, superficial layer of the SC; SCi, intermediate layer of the SC; SCd, deep layer of the SC. Values are mean ± SD for *n* = 5 mice and computed independently of the luminance contrast.

Several trends can be observed when plotting the proportion of voxels attributed to each cluster in function of the luminance contrast. In general, the increase in “high activity” clusters (c3–c5) is concurrent with a decrease in the proportion of c1–c2 voxels (Fig. 4*d*). Clusters c3–c5 do appear once a plateau stage is reached by the previous highest activity cluster (e.g., the increase in the proportion of voxels attributed to c4 follows a plateau in the proportion of c3).

In SCs, a decrease in the proportion of c3 can only be observed from 10% contrast onward, together with the gradual emergence of c5 (Fig. 4*d*). The latter is present in low proportions in SCi only from 50% contrast and absent in SCd. Overall, the clusters c3–c5 appear progressively with the increase in luminance contrast in SCs and extend first to SCi and then to SCd.

The conclusions of the single-voxel clustering are similar to those of the correlation-based analysis, with additional refinements: (1) there is a clear and specific spatial distribution of the activity as the voxels from the same cluster are being closely grouped; (2) the increase of activity among already active voxels is not homogeneous: it is a mix of c3–c5 voxels, forming a spatial gradient from SCs to SCd; and (3) the highest activity is located in SCs.

### Low-contrast comparison

Toward a more direct comparison between region-based averaging, correlation-based analysis, and single-voxel clustering, we investigated the capabilities of each approach to report activity at low luminance contrast, between 1 and 5%. These contrasts elicit sparse, localized responses and thus provide an appropriate context for assessing the relevance of fine-grained spatial information.

**Figure 5.**
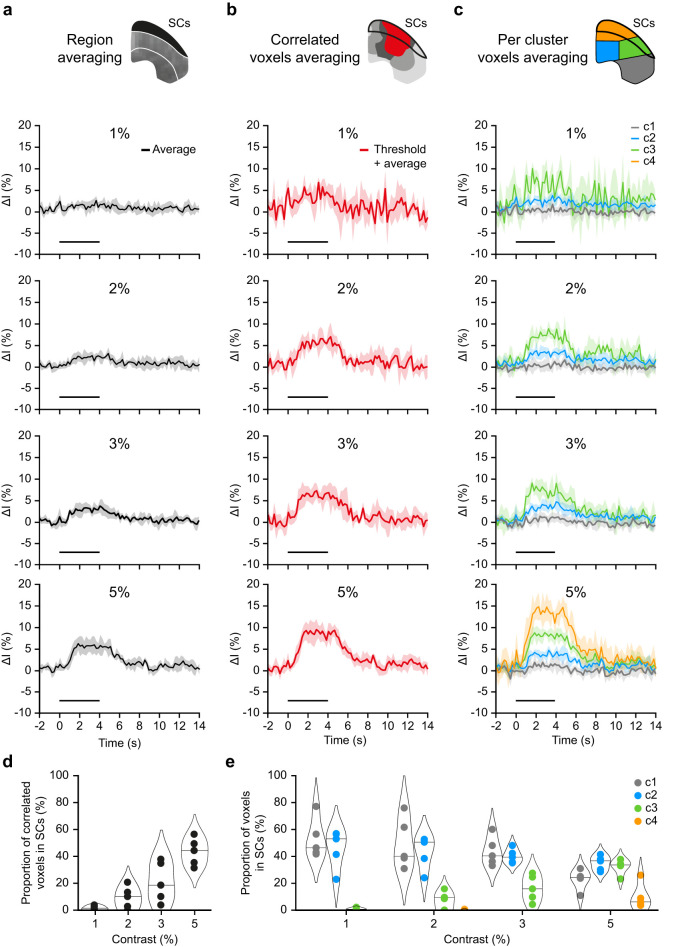
Methods comparison in low-contrast conditions. ***a***, Temporal traces obtained from the averaging of all voxels in the SCs; ***b***, the significantly activated voxels with *R*^2^ > 0.327; ***c***, the voxels based on their cluster attribution at 1, 2, 3, and 5% luminance contrast (top to bottom). Error bands denote the standard deviation. The stimulus period (0–4 s) is depicted by the horizontal black line. ***d***, Change in the proportion of voxels with *R*^2^ > 0.327 and (***e***) per cluster in the SCs, in function of the luminance contrast. Each point denotes a mouse and the horizontal line the median value. The contour represents the kernel density estimation of the underlying samples distribution.

Figure 5*a–c* displays the temporal traces in the SCs, respectively, resulting from the full region, significantly correlated voxels and per-cluster averaging. When comparing the average signal in the SCs with the signals extracted from the clusters, the attenuation of the amplitude caused by the region-based averaging procedure turns prominent. Due to the presence of cluster c3 (at >2% luminance contrast), a peak amplitude of ∼10% is reached compared with the 3–4% luminance contrast observed on the averaged SCs temporal trace (Table 3). This difference in peak amplitude is even larger at 5% contrast with the appearance of cluster c4, bringing the peak amplitude to 17.2 ± 1.6% whereas region-based averaging results in 6.9 ± 1.7%.

**Table 3.** Peak amplitude at low luminance contrast

Cluster	Contrast			
	1%	2%	3%	5%
c1	2.8 ± 1.0	2.5 ± 0.4	2.7 ± 0.5	3.2 ± 0.5
c2	4.4 ± 0.7	5.5 ± 0.8	5.1 ± 0.8	5.3 ± 0.4
c3	11.9 ± 1.7	10.0 ± 0.8	10.8 ± 0.6	10.1 ± 1.1
c4	X	X	X	17.2 ± 1.6
SCs	3.3 ± 1.2	3.6 ± 0.9	4.4 ± 0.9	6.9 ± 1.7

Peak amplitude values computed on the average signal per cluster (c1–c4) and in the entire superficial layer of the SC (SCs). Values are mean ± SD for *n* = 5 mice.

On the contrary, the peak amplitudes measured with the correlation-based voxel selection are consistent with the temporal traces of cluster c3 (e.g., at 5% contrast, *p* = 0.622, *t* test of related samples). Nevertheless, a large underestimation of the amplitude was observed at 5% contrast, corresponding to the first occurrence of voxels assigned to cluster c4 (*p* < 0.01, *t* test of related samples). This discrepancy is explained by the definition of significantly correlated voxels, which does not distinguish between activity levels.

The spatial quantification allowed by the correlation-based analysis exhibits a nearly linear increase in the proportion of correlated voxels within the SCs (Fig. 5*d*). On the other hand, the single-voxel clustering displayed several trends: the increase in the proportion of cluster c3 is nearly linear and compensated by a decrease in proportion of cluster c1, while the proportion in cluster c2 remains broadly constant. Finally, the cluster c4 appears at 5% only in low proportions while cluster c5 is absent.

## Discussion

In this work we explored the suitability of single-voxel clustering as an alternative methodology to analyze fUS data. We made a direct comparison with two conventional techniques, region-based averaging (Macé et al., 2018; Bergel et al., 2020; Brunner et al., 2020, 2021; Sans-Dublanc et al., 2021; Takahashi et al., 2021) and correlation-based analysis (Urban et al., 2014, 2015; Gesnik et al., 2017; Rungta et al., 2017; Rau et al., 2018; Soloukey et al., 2019; Brunner et al., 2021), and evaluated their ability to report spatial and temporal information elicited in the SC by a full-field visual stimulus.

In general, the single-voxel clustering provides a more comprehensive view of the activity than region-based averaging and correlation-based analysis. The spatial dynamics are well represented by the cluster switches, which reveal several trends that were not picked up with the correlation-based quantification and absent upon region-based averaging. The mix of different signals, deriving from nonactive and active voxels, is prominent when averaging. It results in a substantial underestimation of the activity which makes its detection challenging at a low luminance contrast. On the contrary, the correlation maps accurately highlighted the activity between 1 and 3% luminance contrast. The underestimation starts from 5% luminance contrast but to a lower extent than the region-based averaging. Indeed, the use of a threshold does not allow to distinguish different levels of activity, contrary to the single-voxel clustering. Importantly, single-voxel clustering of hundreds of trials can be performed in just a few minutes on a standard computer with sufficient memory (single CPU, 32 GB RAM). For this manuscript, the dataset size was ∼24 GB and was processed in 3 min and 30 s. The slowest step in the analysis is preprocessing, which involves (1) manual registration performed individually for each animal and (2) averaging across trials, experimental sessions, and/or animals. To further streamline this process, we have recently integrated all these functions into an open-source package available on GitHub, which includes full documentation and example datasets (refer to the Materials and Methods section).

A potential issue arising from the use of single-voxel clustering lies in the reproducibility as (1) setting the number of clusters, (2) selecting or not a feature extraction procedure, and (3) choosing the clustering algorithm itself will influence to some extent the readout (Venkatkumar and Shardaben, 2016; Rodriguez et al., 2019; Hancer et al., 2020). Nevertheless, we believe that our approach can be confidently used by any nonexpert scientist by adhering to a set of standard qualitative criteria, including but not limited to (1) validating that individual signals within each cluster exhibit consistent patterns, thereby ensuring that latent clusters have not been unintentionally merged during the process, and (2) verifying the number of clusters to confirm that they are sufficiently distinct from one another. Such a quality check is also essential before any interpretation because the process will potentially aggregate outliers with meaningful signals, what can be problematic when a limited number of voxels are active.

Nevertheless, its paradigm independence as well as spatial resolution make the clustering a powerful tool for data exploration and refining conclusions based on other analyses. The choice of one strategy over another is highly context dependent. When no atlas is available (e.g., neonates; Demene et al., 2016, 2017) or if registration is challenging (e.g., nonhuman primates; Norman et al., 2021), region averaging is not relevant as compared with voxel-based analysis. Likewise, in paradigms with uncertainty about the timing of the activity (e.g., behavioral tasks), computing the correlation based on experimental events is a risky option as part of the activity might be overlooked (Li et al., 2017; Allen et al., 2019; Musall et al., 2019). Toward brain decoding applications, the combination of high spatial resolution with accurate information on HR makes single-voxel clustering a very suitable methodology for selecting an appropriate subset of voxels.

On the other hand, these results illustrate the trade-off between comprehensiveness and dimensionality of the analysis. Indeed, there is a gradient in spatial resolution between the three methodologies, from low (region-based averaging) to high (single-voxel clustering). The conclusion is similar when considering the dimensionality of the outcome: from a region to a binary map to a cluster map, with associated temporal traces. In the context of whole-brain imaging, e.g., with volumetric fUS imaging technology (Brunner et al., 2020), interpretation of single-voxel clustering can become challenging due to the high number of voxels, while region-based averaging can provide a much more convenient visualization of the recorded activity. In such a setting, the recommended way of proceeding would be to first apply the region-based averaging procedure to obtain a general picture of the activity. Further refinement of the analysis could be achieved by applying single-voxel clustering to a defined subset of areas, selected based on a statistical threshold for amplitude levels (e.g., using a *Z*-score). This may be particularly relevant for large regions such as the striatum, hippocampus, or large cortical regions.

When working at the single-voxel scale, assessing the relevance of the extracted information is essential. In this regard, we were able to detect clear low-contrast activity in individual mouse, demonstrating the high sensitivity of the fUS signals outside of the whisker system (Urban et al., 2015; Brunner et al., 2020) and the importance of accurate positioning strategies for detecting subtle HR (Nouhoum et al., 2021; Lambert et al., 2024). Furthermore, the spatial localization of activity is consistent with the functional organization of the SC since the most active clusters were found in the superficial layers which receive the strongest visual input (Basso and May, 2017; Benavidez et al., 2021).

Yet, it should be mentioned that some variability in HR distribution was observed between mice. In particular, the spatial extent of the “highly active” area (clusters c4–c5) varied from remaining confined to the superficial layer to extending downward to the intermediate layer. These differences could partly result from the registration process, which is inherently prone to small shifts, or from a spread of the HR (Sheth et al., 2005; Poplawsky et al., 2019) that could fit with the orientation of vessels supplying the SC from the posterior cerebral artery (Xiong et al., 2017). An alternative explanation is that such activity in the SCi reflects, at least partially, the actual neuronal activity. This latter interpretation is supported by the structured spatial organization of the most active voxels, tightly connected in small groups with like-shaped HR. Indeed, the distribution of hemodynamic responses across animals can significantly impact group-level conclusions due to interanimal variability in vascular and neurovascular dynamics. Such variability can introduce biases or inaccuracies when interpreting brain activity, particularly when pooling data from multiple animals, as in the case of the SC. Differences may arise in the amplitude and temporal dynamics of the responses, as well as their spatial distribution, often influenced by physiological and experimental factors. These challenges can be mitigated through careful physiological monitoring (e.g., heart rate, blood pressure, arousal and stress levels, and other factors) and the use of standardized experimental conditions (Brunner et al., 2021). Further investigations are required for determining the extent to which such small groups of voxels and associated HR can inform us about the underlying local neuronal computations.

## Synthesis

Reviewing Editor: Arvind Kumar, KTH Royal Institute of Technology Decisions are customarily a result of the Reviewing Editor and the peer reviewers coming together and discussing their recommendations until a consensus is reached. When revisions are invited, a fact-based synthesis statement explaining their decision and outlining what is needed to prepare a revision will be listed below. The following reviewer(s) agreed to reveal their identity: Daniel Hillier.

Synthesis

In this manuscript authors present a new method to analyse functional ultrasound data. The methods allows for more precise and detailed activity mapping, which can better highlight specific brain responses. They demonstrate this by estimating low-contrast visual responses in the SC-a relatively underexplored area. Both reviewers agree on the importance of the work for the field of fUS. However there are a few points that need to be clarified. These are mostly related to further elaboration of the data analysis, for instance showing data for individual animals as opposed to the across animal average.

Detailed comments are appended below to help revise the manuscript. We look forward to the revised version.

Reviewer #1

Review of "Spatiotemporal Clustering of Functional Ultrasound Signals at the Single-Voxel Level"

This manuscript introduces a new method, single-voxel clustering, for analyzing functional ultrasound (fUS) imaging data, which tracks brain activity with high detail and broad coverage. Traditionally, fUS data is analyzed through region-based averaging or by correlating it with experimental events, but these methods can be limiting and biased. The proposed method allows for more precise and detailed activity mapping, which can better highlight specific brain responses. It complements existing techniques and is especially useful for analyzing complex brain activity, such as in freely moving subjects or when decoding brain patterns.

The manuscript is clearly written, the figures are very illustrative and to-the-point, and the message is effectively conveyed. The three analysis methods are thoroughly executed. I appreciate the authors' effort to preserve the original quality and sensitivity of the raw data. It is evident that valuable information is lost when using averaging techniques, such as the ROI-based method, or correlation-based approaches. Notably, the single-voxel clustering method retains sensitivity, even for stimuli with very low contrast (as low as 1%), which is not the case for the two alternative methods.

A key limitation, which the authors also note, is the interpretation of the various clusters obtained with the single voxel clustering methos. While not identical, the single-voxel clustering method somewhat aligns with multi-voxel pattern analysis (MVPA), a widely used non-parametric approach in the fMRI community -it would be interesting to adopt that methodology in the future, although I consider it beyond the scope of the present manuscript.

Overall, this manuscript makes a significant contribution to the fUS research field, and I recommend its publication pending the resolution of a few mainly minor issues, which I will outline below.

Major Questions:

1. Averaging across animals: The data in the manuscript are averaged across animals, but it would be ideal to see data for individual animals, especially for the single-voxel clustering analysis. Alternatively, the authors should consider performing an analysis that demonstrates the robustness and reproducibility of the clusters, ideally assessing both within-subject and across-subject reproducibility.

2. Contrast response function (CRF) for the clustering method: While the contrast response function (CRF) is provided for the ROI and correlational analysis, it is not explicitly presented for the clustering method; it is only implied. Could you add the CRF independently for the 5 clusters in Figure 4? Specifically, instead of plotting the proportion of voxels as a function of contrast, the peak amplitude as a function of contrast should be shown for each individual cluster. This would provide insight into which cluster contributes the most to the changes in response as a function of contrast, similar to the other methods presented.

Minor Questions/Suggestions:

1. Line 47: Please clarify what is meant by "non-aligned behavior." Does this refer to decision-making, pre-motor activity, higher-order cognitive control signals, or something else?

2. Line 52: Typo: "which" should be changed to "who."

3. Line 103: Typo: "Once deep anesthesia achieved" should be "Once deep anesthesia was achieved."

4. Line 65: Please clarify what is meant by "fUS studies have used unique or paired voxels for control." Is this referring to specific types of voxels used in experimental design?

5. Line 75: The phrase "by modulating the contrast intensity of the stimulus" could be rephrased for clarity to "by modulating the luminance contrast of the stimulus."

6. Line 81: The term "signal sources" should be clarified. What exactly is meant by "signal sources"? Is it equivalent to "activations," what is then the distinction between "signal sources" and "activity layout"? A clearer explanation of these terms would help.

7. Line 150: Typo: "using custom scripts" or "using a custom script."

8. Line 156: The phrase "stimuli were presented on a grey background (50% luminance)" is incorrect. 50% of what? It would be clearer to provide the actual luminance value in cd/m² (candela per square meter).

9. Line 161: contrast values are not expressed in "intensity" (luminance values are), it should be contrast instead of intensity. Additionally, the exact method of defining luminance contrast (e.g., absolute difference in luminance values divided by the average luminance, or Michelson contrast formula) should be specified clearly.

10. Line 182: Could you provide a detailed explanation of Simpson's estimator?

11. Line 183: This sentence is grammatically incorrect. Please revise it for clarity.

12. Line 275: In Figure 3D, there is a dip at low contrasts for the CRF of SCd. Do you have any insights or explanations for this dip?

13. Line 310: What does Figure 3-1 refer to? Please clarify this reference.

14. Line 385: Could you explain what is meant by an "expertise-based refinement layer"?

15. Biological relevance of the 5 clusters: Could you speculate about the biological significance of the 5 clusters identified? Do they represent different sectors of a topographic map (e.g., a retinotopic map), considering that sensitivity to luminance contrast is eccentricity-dependent? Or?

Reviewer #2

This study introduces single-voxel clustering as an analytical methodology for functional ultrasound (fUS) imaging, providing a valuable in-depth comparison with region-based averaging and correlation-based analysis. The work effectively demonstrates the advantages of single-voxel clustering in resolving subtle spatial and temporal activity patterns, particularly at low stimulus contrast levels.

The authors contextualize their findings within the functional organization of the superior colliculus (SC), corroborating results with established anatomical and physiological knowledge. The discussion is well-balanced, acknowledging both strengths and limitations while offering practical recommendations for integrating these methods in diverse research contexts. The whole manuscript is well written and conveys visually clear messages with only a few minor points to be considered.

By highlighting the trade-offs between spatial resolution, dimensionality, and interpretability, the manuscript offers a robust framework to guide methodological choices in fUS data analysis. The identification of layer-specific activity patterns in the SC adds to our understanding of its functional architecture and demonstrates the sensitivity of fUS for detecting subtle hemodynamic responses (HR).

The manuscript raises important points about reproducibility challenges in clustering (e.g., parameter selection, outlier aggregation). Expanding on strategies to mitigate these issues-such as cross-validation or robustness testing-would strengthen the discussion. Similarly, while the variability in HR distribution across animals is well-described, further elaboration on its implications for group-level conclusions would be helpful. For example, could such variability affect interpretations of SC functional organization?

The recommendation to begin with region-based averaging followed by voxel-based analyses is practical. However, expanding on how this workflow could be adapted for whole-brain imaging, where the number of voxels is vast, would be valuable. For instance, could automated region pre-selection using statistical thresholds streamline the process?

While clustering reveals spatially organized HR patterns, providing additional insights into the biological or functional relevance of specific clusters-such as c4-c5 extending to intermediate layers-would enhance interpretability. Speculating on their potential roles in SC computations or linking them to specific neuronal populations could further enrich the discussion.

Including a comparison of computational resources required for the three methods would offer practical insights into scalability.

Please provide link to original data that can reproduce the figures.
