# Supplementary figures and images for: Spatiotemporal Clustering of Functional Ultrasound Signals at the Single-Voxel Level
Source: eNeuro. 2025 Feb 18;12(2):ENEURO.0438-24.2025. doi: 10.1523/ENEURO.0438-24.2025 (PMC11869936; doi:10.1523/ENEURO.0438-24.2025)

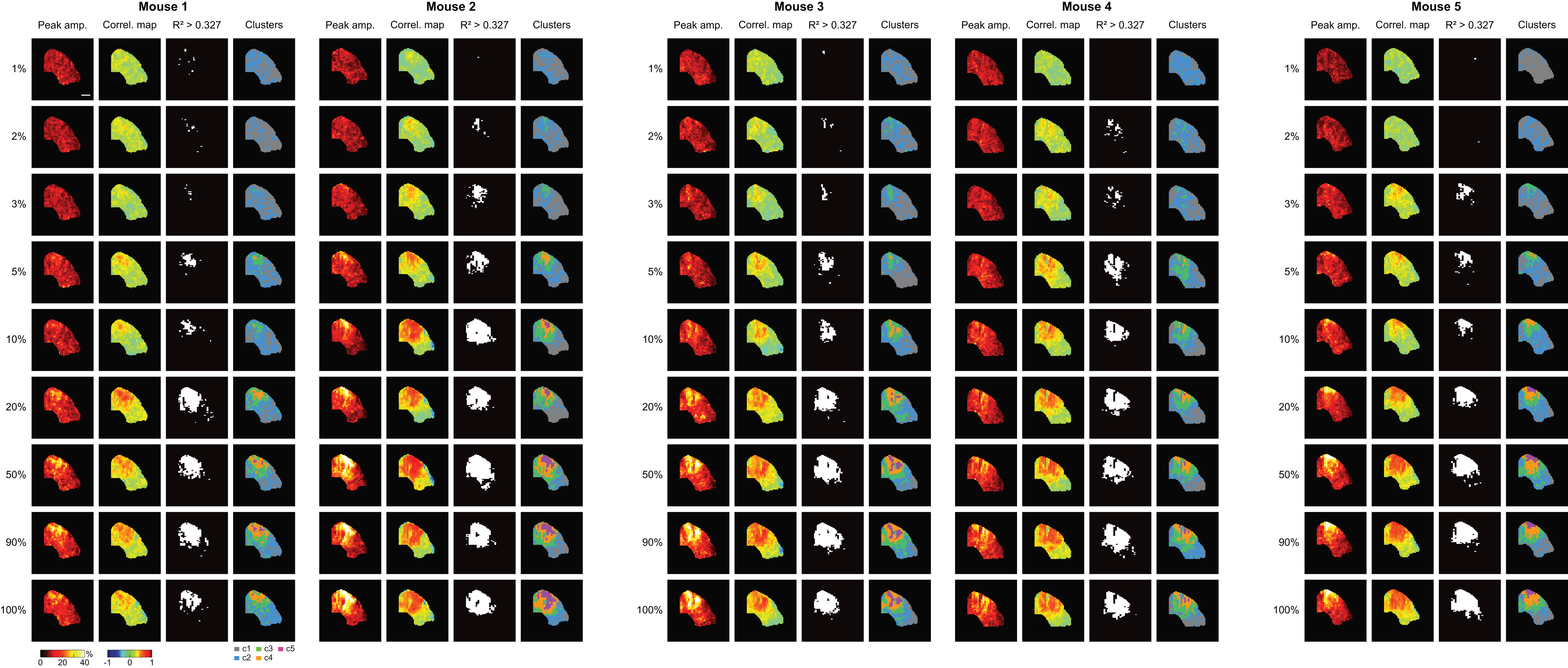

Supplement: Figure 1-1 — Parameters selection for single-voxel clustering. a. Cumulated explained variance ratio of the PCA with respect to the number of principal components. The value 12 was selected, retaining 50% of the explained variance (dashed line). b. Evolution of the inertia with respect to the selected number of clusters. The inflexion point of the curve was used to select K = 5 clusters. Download Figure 1-1, TIF file. [file eneuro-12-ENEURO.0438-24.2025-s003.tif]

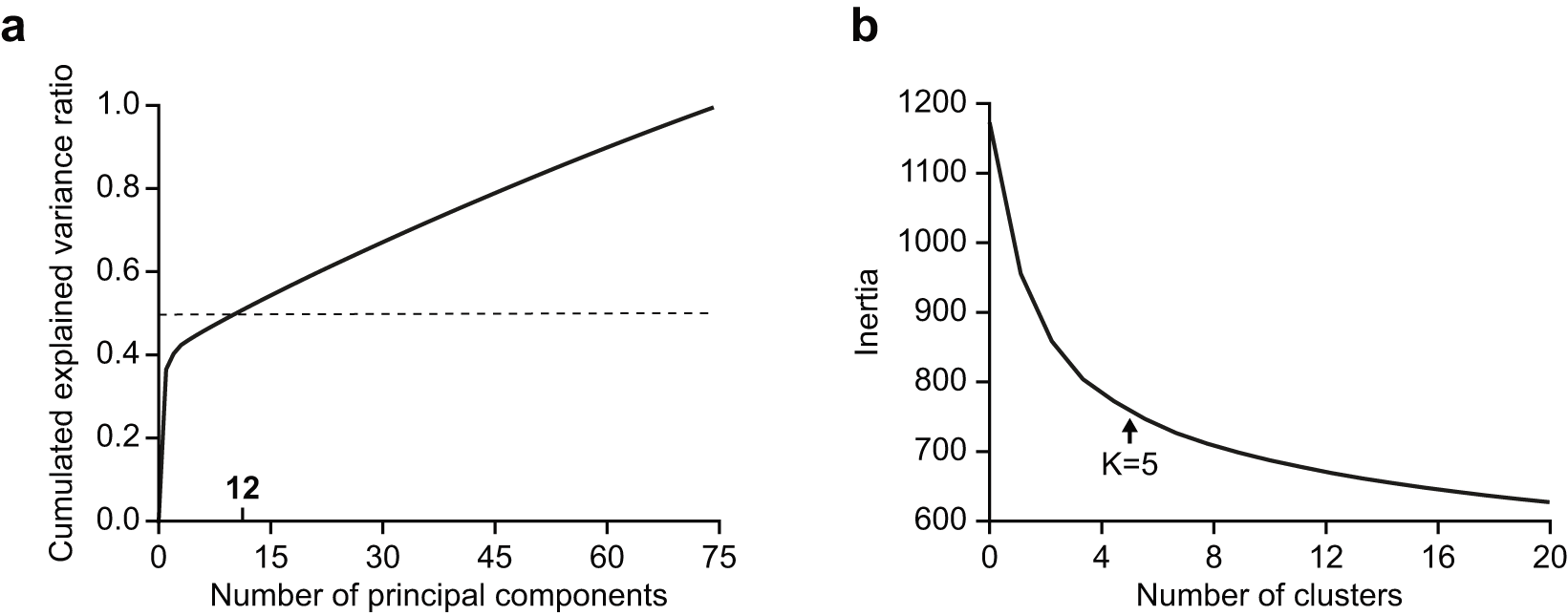

Supplement: Figure 3-1 — Spatial maps across animals and contrasts Spatial maps of the superior colliculus (SC) across all contrast intensities and all the mice (n = 5) used in this work, resulting from peak amplitude computation (left), correlation (center left), correlation with threshold (center right) and single-voxel clustering (right). Scale bar: 0.5 mm. Download Figure 3-1, TIF file. [file eneuro-12-ENEURO.0438-24.2025-s004.tif]
